# Clemastine and hyperthermia enhance sensitization of osteosarcoma cells for apoptosis

**DOI:** 10.1080/23723556.2024.2351622

**Published:** 2024-05-14

**Authors:** Somtochukwu Obu, Suryakant Niture, Hieu Hoang, Sashi Gadi, Vandana, Yiping He, Deepak Kumar

**Affiliations:** a The Julius L. Chambers Biomedical/Biotechnology Research Institute (JLC-BBRI), North Carolina Central University (NCCU), Durham, NC, USA; b Department of Pathology, Duke University Medical Center, Duke University Durham, Durham, NC, USA

**Keywords:** Osteosarcoma, clemastine, hyperthermia, autophagy, 3-methyladenine, cell survival

## Abstract

Clemastine is an antagonist of histamine H1 receptor may provide benefits in the treatment of osteosarcoma (OS). In the current study, we used hyperthermia approach to sensitize OS cells to clemastine-mediated cell death. Osteosarcoma U-2 OS and Saos-2 cells were treated with clemastine at 37°C, followed by 42°C for 2 h, and released at 37°C for 6 h. The impact of clemastine and hyperthermia on OS cell survival and autophagy-mediated cell death was investigated. Exposure of U-2 OS and Saos-2 cells to clemastine and hyperthermia (42°C) inhibited dose-dependent clemastine-mediated cell survival by increasing cell apoptosis. Hyperthermia and clemastine exposure modulated inflammatory and unfolded protein response (UPR) signaling differentially in U-2 OS and Saos-2 cells. Exposure of U-2 OS and Saos-2 cells to hyperthermia and clemastine inhibited AKT/mTOR and induced expression of the autophagy biomarkers LC3B II and LC3-positive puncta formation. The inhibition of autophagy by 3-methyladenine blocked hyperthermia and clemastine-mediated induction of LC3B II, LC3-positive puncta formation, and OS cell apoptosis. These results indicate that clemastine and hyperthermia sensitize OS cell lines by inducing increased autophagic cell death. Collectively, our data suggest that hyperthermia along with antihistamine therapy may provide an improved approach for the treatment of OS.

## Introduction

Osteosarcoma (OS), also known as osteogenic sarcoma, is a cancerous tumor that begins in the bone. OS occurs mainly in children, teens, and young adults. Every year, nearly 1,000 new cases of OS are diagnosed in the United States and nearly 50% of cases are found in children and teens (https://www.cancer.org/cancer/types/OS.html). A worldwide incidence of OS in 3.4 per million people per year has been reported.^1^ Initially, osteosarcoma (OS) is derived from primitive bone-forming mesenchymal cells. OS tends to occur at the sites of bone growth, particularly affecting regions around the knee. The early signs of OS are usually detected by X-rays taken during routine dental checkups. If the OS does not metastasize, survival rates are about 70%, but if it spreads to other parts of the body, the survival rate may be as low as 30%–50%. OS is one of the cancers that can be treated with hyperthermia (heating the lesion site at 42°C) in combination with other therapies such as chemotherapy or radiation therapy. With adjuvant chemotherapies, the survival rate of OS increases from >50 to 65%.^2,3^

Hyperthermia is known to treat cancer tumors.^4^ At a mild temperature increase (37°C to 41°C), local vascular dilation and perfusion increase to enhance oxygen supply to tumors and deep tissue hyperemia that leads to tumor progression.^4^ At hyperthermic conditions (at or above 42°C), there is an increased vascular permeability, allowing the accumulation of protein and fluid in the microenvironment that leads to increased interstitial fluid pressure, resulting in vessel compression and reduction of vascular perfusion. Hyperthermia can induce mechanisms of vascular injury that affect tumor growth and proliferation.^5^ Under hyperthermia conditions, several changes in tumors can occur such as increased membrane fluidity which affects membrane permeability, cytoskeleton changes, and intracellular signal transduction,^6^ which leads to inhibition of tumor growth and metastasis.^5,7^ Earlier studies indicated that certain drugs can also induce hyperthermia and drug-induced hyperthermia may promote an inflammatory syndrome best described as heat stroke^8^ and lead to three of the most widely studied drug-induced hyperthermic syndromes: malignant hyperthermia (MH), neuroleptic malignant syndrome (NMS), and serotonin syndrome (SS), as reviewed recently.^8^

Various reports suggest that hyperthermia-mediated treatment in combination with chemotherapy is effective on various cancers. In one study,^9^ breast cancer cells (MCF-7) were monitored by a metallic culture surface that could evaluate cellular reactions over a range of temperature stimuli, which makes it easier for the hyperthermia treatment to be accurately controlled. This would provide a suitable temperature for an effective hyperthermia treatment.^9^ Maduabuchi et al.^10^ analyzed the effect of hyperthermia at 43°C or 47°C for 1 h on pancreatic adenocarcinoma cells. The study indicated that hyperthermic treatment (under defined environmental conditions) affects pancreatic adenocarcinoma cell-to-cell contact and oxygen status and increases endothelial sprouting by HIF-1α and VEGF secretion which play a role in the inhibition of pancreatic adenocarcinoma.^10^ In another study, it was observed that the hyperthermia treatment could potentially increase the clinical efficacy of gemcitabine (GEM)-based chemotherapy on pancreatic cancer cells (PANC-1 and ASPC-1).^11^ The results from this study showed that pre-conditioning the cells to hyperthermia (43°C for 60 min) before treating with GEM treatment yielded a superior effect as compared to other treatment regimens. This combination therapy in comparison to GEM only showed better results by decreasing cell viability and promoting cell apoptosis through the induction of excessive ROS production.^11^

Hyperthermia is used to treat OS.^12,13^ Along with chemotherapy or radiation therapy, hyperthermia is used to treat post-actinic bone tumors.^13^ Hyperthermia increases drug cytotoxicity in tumors because of the high fluidity of phospholipid bilayers in tumor cells, which enhances drug permeability.^7,14^ Hyperthermia is an effective local-adjuvant therapy for carcinomas and sarcomas. A randomized phase III trial showed that regional hyperthermia combined with neo-adjuvant chemotherapy for soft tissue sarcomas had better local progression-free survival than chemotherapy alone.^15^ Several reports have suggested that hyperthermia for OS achieved an effective response, including the induction of apoptosis^16^ inhibition of tumor proliferation,^17^ and DNA synthesis *in vitro*.^18^ Human OS (U-2 OS) cells, when exposed to hyperthermia, increased endoplasmic reticulum stress and production of reactive oxygen species that lead to induced cell apoptosis.^19^ Earlier study suggests that hyperthermia reduces OS cell migration by suppression of autocrine motility factor (AMF).^20^ Regional hyperthermia using an alternating magnetic field reduced the pulmonary metastasis of OS in an *in vivo* study.^21^ Moreover, a recent review indicated that effective drug delivery systems are using nanoparticles and mild hyperthermia in solid tumor therapy.^22^

Clemastine fumarate is a selective histamine H1 receptor antagonist.^23^ Clemastine, due to its anti-histamine activity, is used to relieve allergies, runny nose, hay fever, sneezing, red/itchy/tearing eyes, and swelling of hives (https://www.ncbi.nlm.nih.gov/books/NBK548709/). Histamine receptors are known to be involved in tumor progression in various cancers.^24^ A recent study suggested that increased expression of histamine receptor H1 is associated with both recurrence-free survival and overall survival in liver cancer patients.^25^ High expression of histamine receptor H1 in SNU-368 hepatocellular carcinoma (HCC) cells increased HCC cell cycle progression, growth, and metastasis by suppression of cell apoptosis, whereas inactivation of histamine receptor H1 by terfenadine suppressed HCC tumor growth and metastasis.^25^

In the present study, we examined the sensitivity of clemastine on OS U-2 OS and Saos-2 cells under normal and hyperthermic conditions. Our data suggest that exposure of OS cells to clemastine and hyperthermic conditions triggers autophagic cell death/apoptosis.

## Results

### Hyperthermia affects clemastine-mediated OS cell survival

**Figure 1.**
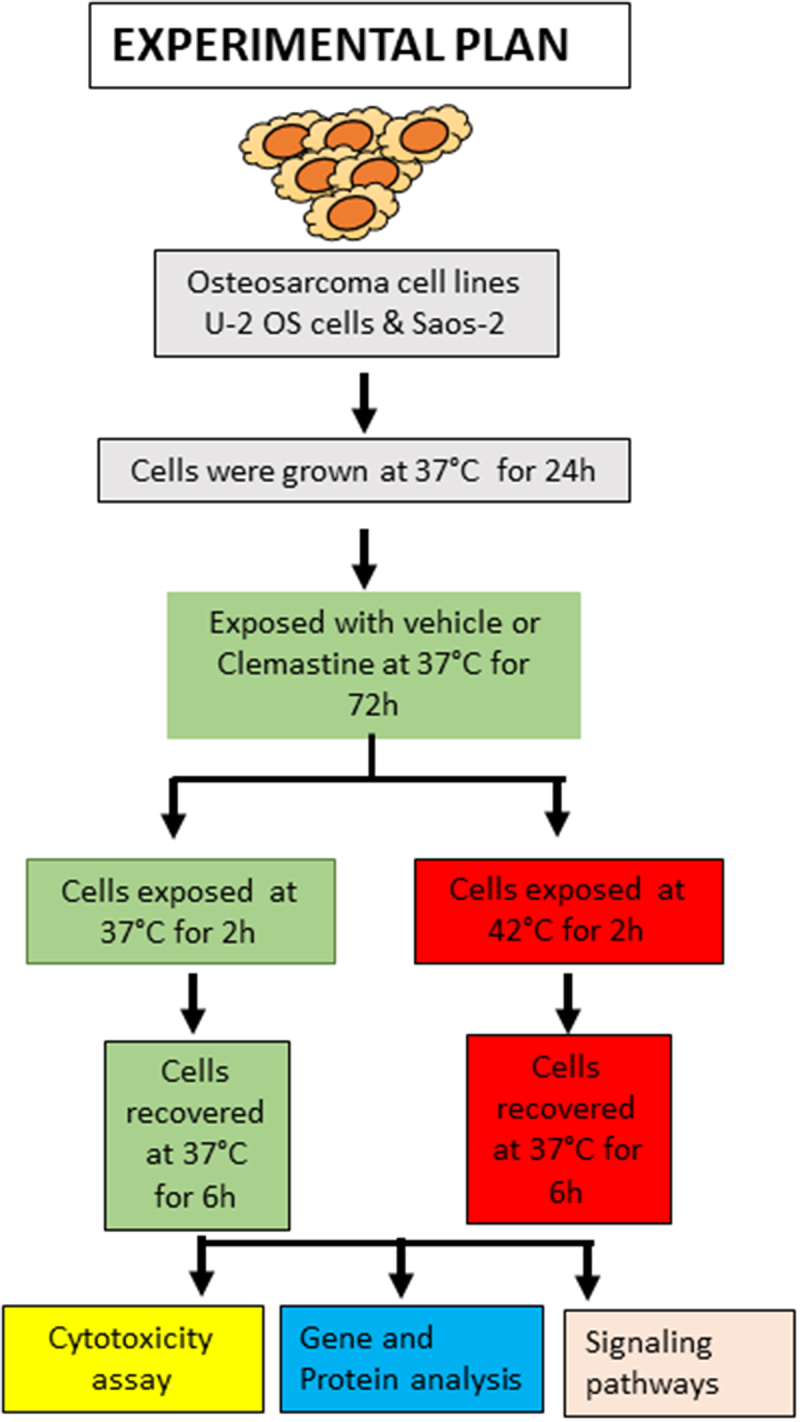
Schematic diagram of the experimental plan. The osteosarcoma cell lines (U2- OS and Saos-2) were cultured at 37°C for 24 h and then exposed to vehicle/clemastine at 37°C for 72 h. Another set of cells were exposed to hyperthermia at 42°C for 2 h and returned to 37°C for a 6 h recovery phase. The expression of multiple signaling pathway markers was analyzed after treatment.

Treatment of OS is challenging and new approaches are urgently needed.^26^ Here, we used a unique approach to sensitize bone sarcomas such as OS cells to hyperthermia, in the presence of the histamine H1 receptor antagonist clemastine. As shown in Figure 1, OS cells U-2 OS and Saos-2 cells were treated with clemastine at 37°C, followed by 42°C for 2 h for hyperthermic conditions, and released at 37°C for 6 h. As a control, cells were also incubated without hyperthermic conditions at 37°C ± clemastine (Figure 1). After the application of hyperthermic conditions, cells were released for 6 h at 37°C and analyzed for alterations in cellular and biochemical pathways to determine the comparative sensitivity of clemastine exposure to OS cells under hyperthermic and normal conditions.

**Figure 2.**
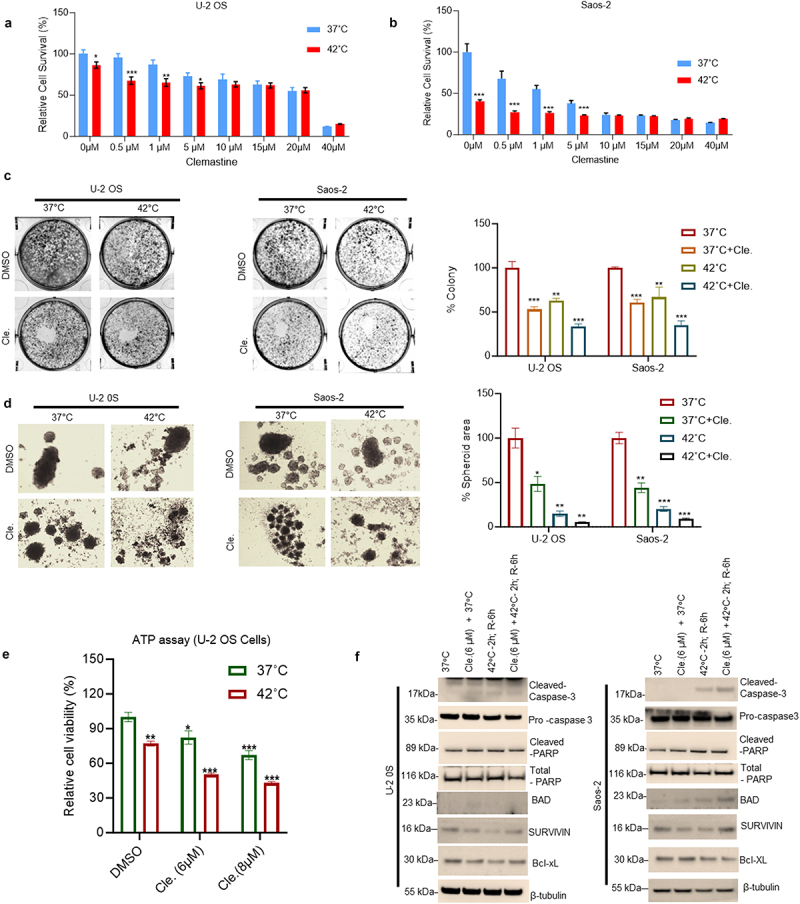
Hyperthermia inhibits clemastine-mediated OS cell survival. A & B) U-2 OS and Saos-2 cells were plated in 96 well plates at a density of 3000 cells/well for 24 h and treated with 0.5–40 µM clemastine for 72 h. The hyperthermic treatment was performed in a 42°C incubator for 2 h and returned to a 37°C incubator for 6 h recovery phase. A cell viability MTT assay was performed as described in the materials and methods section. The experiment was repeated thrice. *p < .05, **p < .01, ***p < .001 compared to the vehicle untreated cells. C) The cell colony formation assay. U-2 OS and Saos-2 cells (10,000 cells/well) were treated with clemastine or clemastine + hyperthermia as indicated, and a cell colony formation assay was performed. The blue colonies were photographed and counted manually, and the percentage of colony formation was plotted. **p < .01, ***p < .001 compared to the vehicle untreated cells. D) U-2 OS and Saos-2 cells (10,000 cells/well) were grown in ultra-low attachment surface 24 well plate (Corning) for 24 h and cells were treated with clemastine or clemastine + hyperthermia as indicated and cell spheroid formation was monitored, and images were captured under a light microscope using 4X objective (left panels). The spheroids area (*n* = 3) was calculated using ImageJ software (https://imagej.nih.gov/ij/) and plotted (right panels). ***p  <  .001, **p   < .01 *p<.05 compared to control cells. E) U-2 OS and Saos-2 cells were treated with 6 µM and 8 µM clemastine for 72 h. The hyperthermic treatment was performed in a 42°C incubator for 2 h and returned to a 37°C incubator for 6 h recovery phases. A cell viability ATP assay was performed as described in the materials and methods section. The experiment was repeated thrice *p < .05, **p < .01, ***p < .001 compared to the untreated/vehicle cells. F) U-2 OS and Saos-2 cells were treated with 6µM clemastine for 72 h and the hyperthermic treatment was performed in a 42°C incubator for 2 h and returned to 37°C incubator for 6 h. The expressions of cleaved caspase-3, pro-caspase-3, cleaved-PARP, BAD, survivin, Bcl-xL, and β-tubulin were analyzed by immunoblotting.

To examine how hyperthermia affects clemastine-mediated OS cell survival, we performed MTT assays, and cell metabolic activities/cell survival were measured. Both U-2 OS and Saos-2 cells treated with increasing concentrations of clemastine (0.5 µM–40 µM) at 37°C for 72 h showed a dose-dependent significant decrease in cell survival (Figure 1a, b). Interestingly, when clemastine-treated cells were exposed to 42°C for 2 h followed by 37°C for 6 h, there was a further decrease in cell survival (~20–35%) compared with 37°C exposed and clemastine-treated cells (Figure 1a, b), suggesting that acute hyperthermia exposure increases clemastine sensitivity in OS cells. We also quantified the live cell number after exposure to clemastine (6 µM) and hyperthermia by trypan blue cell viability assay. Our data suggest that exposure to clemastine and hyperthermia decreased cell viability (~26% in U-2 OS and ~19% in Saos-2) when compared with clemastine-treated cells exposed at 37°C (Supplementary Figure S1). Next, we analyzed the impact of clemastine and hyperthermia on the cell colony formation ability of OS cells (Figure 2c). U-2 OS and Saos-2 cells were treated with clemastine (6 µM) for 72 h, exposed to hyperthermia for 2 h, and released at 37°C for 72 h. Clemastine treatment decreased colony formation by 47% and 40% at 37°C compared with vehicle-treated U-2 OS and Saos-2 cells, respectively (Figure 2c, left and right panels). Hyperthermia exposure (42°C for 2 h) decreased colony formation by 37.1% and 33.2% compared with 37°C exposure and vehicle-treated U-2 OS and Saos-2 cells, respectively. Clemastine treatments and hyperthermia exposure further decreased cell colony formation by 66.6% and 64.63% compared with 37°C exposure and vehicle-treated U-2 OS and Saos-2 cells, respectively (Figure 2c, left and right panels). Similarly, clemastine treatment decreased U-2 OS and Saos-2 cell spheroid formation (~51.6% and ~55.9%) and clemastine + hyperthermia exposure further decreased U-2 OS and Saos-2 cell spheroid formation (~94.9% and ~91.18%) *in vitro* compared with 37°C exposure and vehicle-treated U-2 OS and Saos-2 cells, respectively (Figure 2d, left and right panels).

We also analyzed endogenous ATP production after clemastine + hyperthermia exposure in U-2 OS cells. Our data suggest that clemastine treatment decreased ATP production (32.9%) in U-2 OS at 37°C and that clemastine + hyperthermia exposure further decreased ATP production (57.14%) in U-2 OS compared with 37°C exposure and vehicle-treated U-2 OS cells (Figure 2e). In addition, we analyzed oxygen consumption rate (OCR) after exposure of OS cells with clemastine and clemastine + hyperthermia. Our data suggest that clemastine + hyperthermia exposure affects mitochondrial OCR in both cell lines (Supplementary Figure S2). These results indicate that clemastine + hyperthermia exposure affects mitochondrial function in OS cells. We further analyzed the effect of clemastine (6 µM) and clemastine + hyperthermia on the expression of anti-apoptotic and apoptotic markers by immunoblotting (Figure 2f). The immunoblotting data demonstrated that clemastine + hyperthermia exposure increased expression of cleaved-caspase 3 in U-2 OS and Saos-2 cells significantly, and no significant cleaved-PARP expression was observed when cells were exposed to clemastine + hyperthermia (Figure 2f and supplementary Figure S3). Clemastine + hyperthermia also increased BAD expression in Saos-2 cells. Clemastine or hyperthermia exposure also decreased the expression of Survivin and Bcl-xL in both cell lines (not significantly) compared to control cells exposed at 37 °C (Figure 2f). Collectively, our data suggest that clemastine + hyperthermia exposure affects OS cell survival and cell apoptosis.

### Clemastine and hyperthermia exposure modulates inflammatory and UPR signaling in OS cells

**Figure 3.**
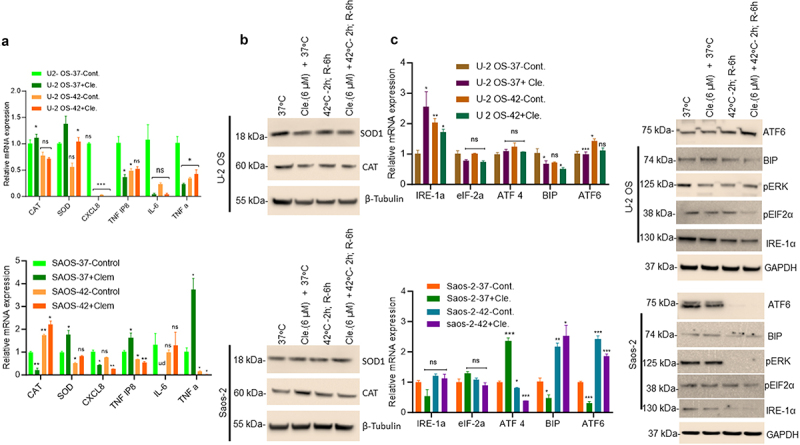
Clemastine and hyperthermia exposure regulates inflammatory and UPR signaling. A) U-2 OS and Saos-2 cells were treated with 6 µM clemastine for 72 h. The hyperthermic treatment was performed as described in the methods section. The relative mRNA expression of *CAT*, *SOD1*, *CXCL8* (*IL-8*), *TNFAIP8*, *IL-6*, and *TNFα* were analyzed by RT/QPCR as described in the materials and methods section. *p<.05, **p<.01, ***p<.001 compared to vehicle treated OS cells. B) The effect of clemastine and clemastine + hyperthermia on SOD and CAT protein expression in OS cells was analyzed by immunoblotting (upper and lower panels). C) U-2 OS and Saos-2 cells were treated with 6 µM clemastine for 72 h. The hyperthermic treatment was performed as described in the methods section. The relative mRNA expression of indicated UPR genes was analyzed by RT/QPCR as described in the materials and methods section. *p<.05, **p<.01, ***p<.001 compared to vehicle treated OS cells. D) U-2 OS and Saos-2 cells were treated with 6 µM clemastine for 72 h. The clemastine and hyperthermic treatment was performed as indicated and as described in the methods section. Cells were then lysed and 60 µg of cell lysates were immunoblotted with ATF6, BIP, pERK, pEIF2α, IRE-1α, and GAPDH antibodies.

Since clemastine and hyperthermia reduced OS cell survival by induction of cell apoptosis, we ask whether clemastine and hyperthermia modulate the inflammatory response and ER-related unfolded protein response (UPR) signaling. We analyzed the effect of clemastine (6 µM) and clemastine **+** hyperthermia on the expression of inflammatory markers by RT/qPCR and immunoblotting (Figure 3a,b). RT/qPCR data suggest that clemastine exposure increased *CAT* (1.11-fold) and *SOD* (1.38-fold) gene expression in U-2 OS cells compared with vehicle-treated cells. However, the expressions of *CAT*, *SOD1*, *CXCL8*, *TNFα*, *TNFAIP8*, and *IL-6* gene expression significantly decreased in U-2 OS cells when exposed to hyperthermia alone or clemastine and hyperthermia compared with vehicle-treated cells at 37°C (Figure 3a, upper panel). In Saos-2 cells, clemastine exposure increased expression of *SOD* (1.76-fold), *TNFα* (3.74-fold), and the *TNFα* response gene *TNFAIP8* (1.64-fold) compared with vehicle-treated cells. Hyperthermia exposure increased the expression of *CAT* (1.74-fold) and clemastine and hyperthermia exposure increased the expression of *CAT* (2.2-fold) in Saos-2 cells. However, the expression of *SOD1*, *CXCL8*, *TNFα*, and *TNFAIP8* gene expression significantly decreased in Saos-2 cells when the cells were exposed to hyperthermia alone or clemastine and hyperthermia compared with vehicle-treated cells at 37°C (Figure 3a, lower panel). In addition, we also analyzed inflammatory protein expressions such as antioxidant-related superoxide dismutase 1 (SOD1), and catalase (CAT) after exposure to clemastine and hyperthermia in OS cells by immunoblotting. Our data demonstrated that no change in SOD1 and CAT protein expression was observed when OS cells were exposed to clemastine or hyperthermia as indicated (Figure 3b, upper & lower panels).

Further, we analyzed the effects of clemastine or hyperthermia on the expression of five key UPR pathway biomarkers, *IRE1a, eIF2a*, *ATF4*, *BIP, and ATF6*. Exposure to clemastine increased *IRE-1α* (2.55-fold) expression in U-2 OS and *ATF4* (2.36-fold) in Saos-2 cells (Figure 3c). However, exposure to clemastine or clemastine + hyperthermia showed no significant change in expression of *eIF2a*, *ATF4*, *BIP*, and *ATG6* genes in U-2 OS cells. Interestingly, in Saos-2 cells, clemastine upregulated *ATF4* (2.36-fold) expression, hyperthermia-induced expression of *BIP* (2.18-fold) and *ATF6* (2.43-fold) while clemastine + hyperthermia increased expression of *BIP* (2.52-fold) and *ATF6* (1.86-fold) compared to 37°C vehicle-exposed cells (Figure 3c, left panels). Moreover, immunoblotting data suggest that clemastine + hyperthermia decreased expression of IRE1α, pEIF2α, pERK, and BIP in both cell lines compared with vehicle-treated cells at 37°C. ATF6 expression increased in U-2 OS cells, whereas its expression was decreased in Saos-2 cells (Figure 3c, right panels). Our data suggest that clemastine + hyperthermia modulate inflammatory and UPR signaling differentially in U-2 OS and Saos-2 cells.

### Combined exposure to clemastine and hyperthermia inhibits AKT/mTOR and induces cellular autophagy in OS cells

**Figure 4.**
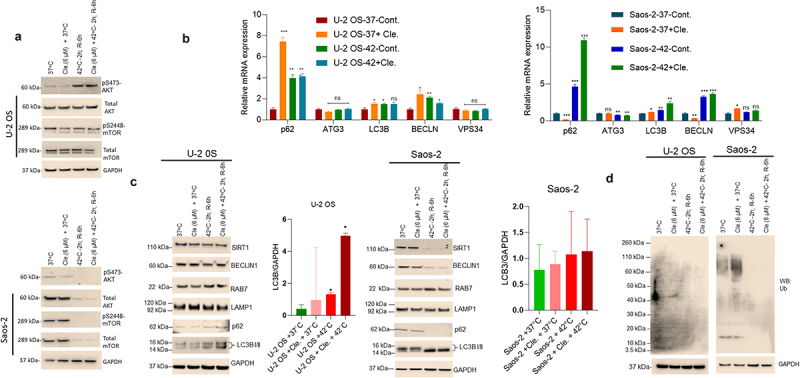
Combined clemastine and hyperthermia exposure inhibits AKT/mTOR and induces cellular autophagy in OS cells. A) U-2 OS and Saos-2 cells were treated with 6 µM clemastine for 72 h. The hyperthermic treatment was performed as described in the methods section. Cells were then lysed and 60 mg of cell lysates were immunoblotted with pS473-AKT, AKT, pS2448-mTOR, mTOR, and GAPDH antibodies (upper and lower panels). B) U-2 OS and Saos-2 cells were treated with 6 µM clemastine for 72 h alone or further exposed to the hyperthermic condition as described in the methods section. The relative mRNA expression of *p62*, *ATG3*, *LC3B*, *BECLIN*, and *VPS-34* were analyzed by RT/qPCR as described in the materials and methods section. *p<.05, **p<.01, ***p<.001 compared to the vehicle treated cells. C) U-2 OS and Saos-2 cells were treated with 6 µM clemastine for 72 h. The hyperthermic treatment was performed as described in the methods section. Cells were then lysed and 60 µg of cell lysates were immunoblotted with autophagy biomarkers such as Sirt1, Beclin1, Rab 7, Lamp1, p62, LC3B I/II, and GAPDH. LC3B I/II and GAPDH band intensities were quantified by image J (https://imagej.nih.gov/ij/) and plotted. Similarly, the lysates were also immunoblotted with anti-ubiquitin mouse antibody (D).

Since clemastine and hyperthermia sensitized OS cells to apoptosis and inhibited cell proliferation, we further analyzed the effect of clemastine and hyperthermia on AKT/mTOR signaling, since the AKT/mTOR signaling pathway is involved in cell survival and proliferation and is activated in various human cancers.^27^ U-2 OS and Saos-2 cells were exposed to clemastine and hyperthermia alone or in combination and expression of AKT/mTOR was analyzed by western blotting. Clemastine treatment alone at 37°C did not show any effect on pS473-AKT and pS2448-mTOR expression in both OS cell lines, however, hyperthermia-induced pS473-AKT in U-2 OS cells and suppressed pS473-AKT and pS2448-mTOR expression in Saos-2 cells and clemastine + hyperthermia exposure downregulated pS473-AKT and pS2448-mTOR in Saos-2 cells (Figure 4a, upper and lower panels). This indicates that clemastine + hyperthermia inhibit AKT/mTOR signaling in OS cells.

Since the activation of AKT/mTOR inhibits autophagy,^28^ we further analyzed the effect of clemastine and hyperthermia alone or in combination on the autophagy gene/protein markers. RT/qPCR data demonstrated that clemastine and hyperthermia alone induced expression of *p62* (7.46 fold and 3.98 fold), *LC3B* (1.53 fold and 1.49 fold), and *BECLN* (2.42 fold and 2.14 fold) in U-2 OS cells, respectively, whereas clemastine in combination with hyperthermia induced expression of *p62* (10.9 fold), *LC3B* (2.40 fold), and *BECLN* (3.65 fold) in Saos-2 cells indicating that clemastine in combination with hyperthermia induced autophagic related gene expression in OS cells (Figure 4b, left and right panels). Immunoblotting data demonstrated that clemastine treatment at 37°C did not influence autophagic flux on OS cell lines since no significant change in the expression of Sirt1, Beclin1, Rab7, Lamp1, and p62 and Lc3b was observed. However, hyperthermia alone or in combination with clemastine increased the expression of LC3b II in OS cells (Figure 4c, left and right panels) suggesting that clemastine in combination with hyperthermia may regulate autophagy in the initial stages. In addition, we analyzed the effect of clemastine and hyperthermia on the cellular ubiquitination of proteins. Our data showed that clemastine alone stimulates ubiquitination at 37°C; however, hyperthermia alone or in combination with clemastine decreased cellular ubiquitination of the proteins in both cell lines, which could be due to the inactivation of ubiquitin activating enzyme E1 (Ub-E1) (Figure 4d, left and right panels). The data suggests that clemastine and hyperthermia may regulate cellular autophagy and sensitize OS cells for apoptosis.

### Inactivation of autophagy impairs sensitization of cell apoptosis modulated by combined exposure to clemastine and hyperthermia in OS cells

**Figure 5.**
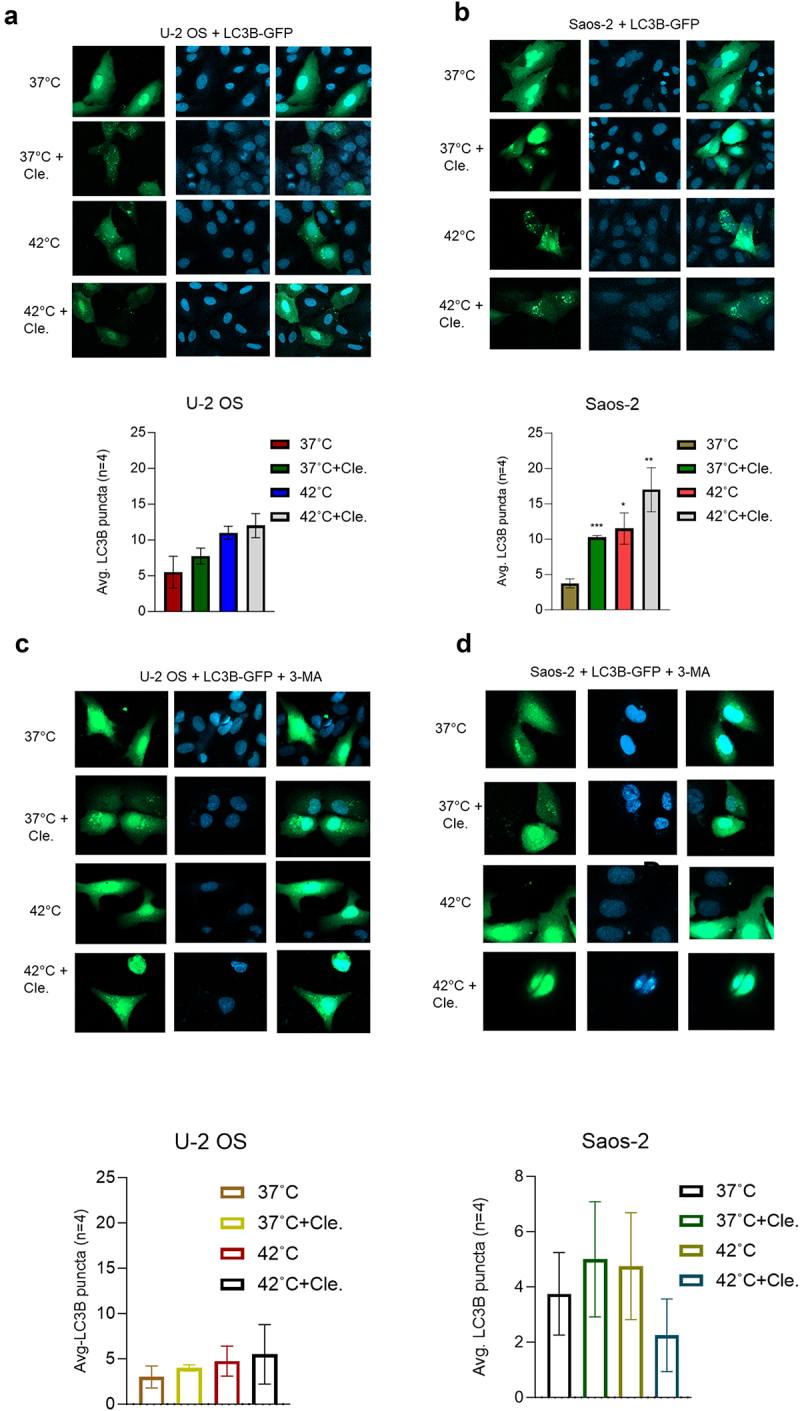
3-MA inhibits LC3B-mediated puncta formation in OS cells after combined exposure to clemastine and hyperthermia. A & B) U-2 OS and Saos-2 cells (1x10^5) were transfected with 1 µg of EGFP-LC3B plasmid DNA and cells were treated with vehicle/6 µM clemastine for 72 h, then 2 h hyperthermic treatment and 6 h recovery phase. Cells were mounted on microscopic slides with a mounting medium containing nuclear DAPI stain. Imaging of the cells was carried out using a confocal fluorescence microscope. The number of GFP-LC3B-related puncta formed in the cells (*n*=4) was counted and plotted *p<.05, **p<.01, ***p<.001 compared to the vehicle-treated cells. C & D). U-2 OS and Saos-2 cells (1x10^5) were plated in 6 well plates for 24 h and transfected with 1 µg of EGFP-LC3B plasmid DNA. Cells were treated with vehicle/6 µM clemastine for 12 h then treated with 2 mM 3-MA for 60 h and exposed to 2 h hyperthermic treatment and 6 h recovery phase. Cells were mounted on microscopic slides with a mounting medium containing nuclear DAPI stain. Imaging of the cells was carried out using a confocal fluorescence microscope. The number of GFP-LC3B-related puncta formed in the cells (*n*=4) was counted and plotted.

To examine whether clemastine and hyperthermia trigger OS cell apoptosis/death through autophagy, we further visualized the induction of LC3B puncta in OS cell lines. For this, U-2 OS and Saos-2 cells were transiently transfected with EGFP-LC3B plasmid for 18 h and cells were exposed to clemastine followed by hyperthermia treatment (Figure 5a,b). Immunocytochemistry images and data suggest that exposure to clemastine or hyperthermia-induced LC3B-puncta formation in both OS cell lines (Figure 5a,b). The combination of clemastine and hyperthermia also stimulated LC3B-puncta formation in both OS cell lines compared with cells transfected with EGFP-LC3B plasmid at 37°C (Figure 5a,b, upper and lower panels). To inhibit cellular autophagy we used 3-methyladenine (3-MA), a class III PI3K inhibitor which is widely used for autophagy inhibition.^29^ To confirm that 3-MA inhibits autophagy, OS cell lines were transfected with EGFP-LC3B plasmid DNA, treated with 3-MA, and exposed to clemastine and hyperthermia as indicated in Figure 5c,d. Pre-treatment with 3-MA was unable to stimulate LC3B-puncta formation in OS cell lines when they were exposed to clemastine and hyperthermia alone or in combination (Figure 5c,d, upper and lower panels) compared with 37°C vehicle-exposed cells.

**Figure 6.**
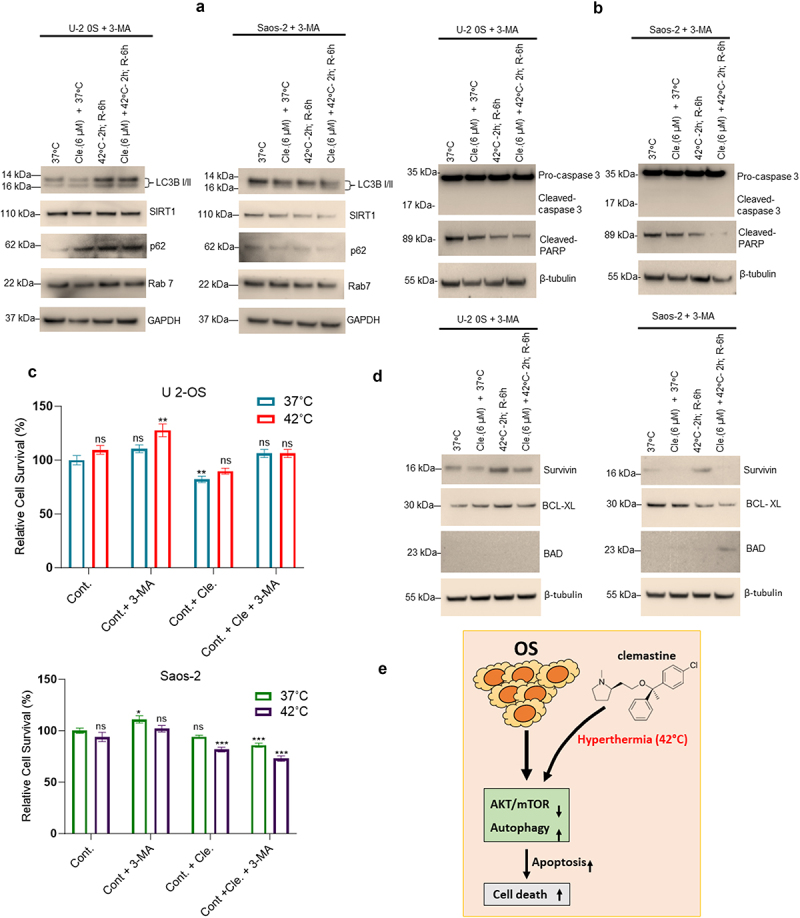
Inactivation of autophagy by 3-MA impairs sensitization of cell apoptosis after combined exposure to clemastine and hyperthermia in OS cells. A & B) U-2 OS and Saos-2 cells were treated with 6 µM clemastine and/or 2 mM 3-methyladenine for 72 h. The hyperthermic treatment was performed in a 42°C incubator for 2 h and then returned to 37°C for a 6 h recovery phase. Cells were then lysed and 60 µg of cell lysates were immunoblotted with Sirt1, p62, LC3B I/II, Rab7, pro-caspase 3, cleaved caspase 3, cleaved-PARP and GAPDH, and β-tubulin antibodies. C) U-2 OS and Saos-2 cells were treated with 6 µM clemastine and/or 3-methyladenine (2 mM) for 72 h. The hyperthermic treatment was performed in a 42°C incubator for 2 h and then returned to 37°C for 6 h recovery phase. Cell viability MTT assay was also performed as described in materials and methods. *p<.05, **p<.01, ***p<.001 compared to the untreated/vehicle treated cells. D) U-2 OS and Saos-2 cells were treated with 6 µM clemastine and/or 2 mM 3-methyladenine for 72 h. The hyperthermic treatment was performed in 42°C incubator for 2 h and then returned to 37°C for a 6 h recovery phase. Cells were then lysed, and 60 µg of cell lysates were immunoblotted with survivin, Bcl-xL, BAD, and β-tubulin antibodies. E) Schematic diagram represents the role of hyperthermia and clemastine in increasing autophagy-mediated cell death.

To test whether clemastine and hyperthermia induce OS cell apoptosis through autophagy, we further analyzed the expression of autophagy, apoptosis, and cell survival biomarker expression in 3-MA pre-treated OS cells after exposure to clemastine and hyperthermia alone or in combination. Immunoblotting data suggest that pre-treatment with autophagy inhibitor 3-MA, clemastine, and hyperthermia alone or in combination were unable to lipidate LC3-B/I into LC3-B/II which indicates the inactivation of cellular autophagy (Figure 6a). No change in expression of Sirt1 and RAB7 was observed in both cell lines, whereas p62 expression increased in U-2 OS cells and no change in expression of p62 was observed in Saos-2 cells compared to 3-MA treated cells at 37°C (Figure 6a, left and right panels). In addition, cleaved-caspase 3 expression was not detected, and cleaved-PARP expression was decreased in OS cell lines when cells were pre-treated with 3-MA and exposed to clemastine and hyperthermia alone or in combination (Figure 6b, left and right panels). We analyzed OS cell survival activities by MTT assay after exposure to clemastine and hyperthermia alone or in combination in 3-MA pre-treated cells as indicated (Figure 6c, upper and lower panels). Exposure with 3-MA or a combination of clemastine and hyperthermia did not show any significant effect on cell survival when compared with 37°C exposed cells (Figure 6c, upper and lower panels). However, pre-treatment with 3-MA and exposure to clemastine did not affect mitochondrial OCR but the combination of clemastine and hyperthermia affected mitochondrial OCR in 3-MA pretreated OS cells (Supplementary Figure S4). Finally, immunoblotting data suggest that pre-treatment-with-3-MA, and exposure to clemastine and hyperthermia in combination increased the expression of cell survival factors. In U2-OS cells, pre-treatment with 3-MA and exposure clemastine + hyperthermia increased Survivin and Bcl-xL and apoptotic BAD expression was not detected compared to 3-MA pre-treated and at 37°C exposed cells (Figure 6d, left panels). In the Saos-2 cells, pre-treatment with 3-MA and hyperthermia increased Survivin expression compared to 3-MA pre-treated and at 37°C exposed cells. However, pre-treatment with 3-MA and clemastine **+** hyperthermia exposure decreased Survivin and Bcl-xL proteins but did not increase BAD expression (Figure 6d, right panels). Collectively, our data suggest that clemastine in combination with hyperthermia enhanced cell apoptosis/death in OS cells by regulation of cellular autophagy.

## Discussion

Osteosarcoma (OS) is a rare bone cancer malignancy (0.2% of all cancers).^30^ The survival rate is very poor in patients when bone cancer tumors recur and metastasize to other organs. OS is a heterogeneous cancer in both its origins and manifestations.^31^ Several factors are associated with OS development such as age, gender, race, and genomic alterations. For early diagnosis of OS, several noninvasive methods are used. For example, determination of alkaline phosphatase levels in serum, X-ray imaging, NMR imaging, computed tomography (CT) scanning, positron emission tomography (PET), biopsy, and microscopic examination are all used for OS diagnosis.^31^ OS is a metastatic disease and, based on the location, is classified as pulmonary metastatic or extrapulmonary metastatic OS disease which is usually lethal.^32^ Metastatic OS is associated with poorer prognoses with a 13% survival rate at 5 y^33^ with about 80% of cases of lung metastasis involved.^34^ If the OS does not metastasize, survival rates are about 70% but if it spreads to other parts of your body, the survival rate may be as low as 30%–50%.

Hyperthermia is used as an effective local-adjuvant therapy for OS and hyperthermia in combination with other therapies such as chemotherapy or radiation therapy is known to increase the survival rate of OS patients (>50 to 65%).^2,3^ In the current study, we used clemastine and hyperthermia to sensitize OS cells for apoptosis. Clemastine is an antagonist of the histamine H1 receptor,^23^ and histamine receptors are known to be involved in tumor progression in various cancers.^24^ However, the role of clemastine in OS is still unknown. Our data suggest that treatment of OS cell lines (U2-OS and Saos-2) resulted in a dose-dependent inhibition of cell survival and the effect of clemastine was further enhanced when clemastine-treated cells were exposed to hyperthermic conditions (42°C for 2 h). Cell colony formation assay and spheroid formation assay suggested that a combination of clemastine and hyperthermia suppressed OS cell growth and also reduced ATP production, and mitochondrial oxygen consumption rate (OCR) suggesting that the effect of clemastine is greater when cells are exposed to acute hyperthermia. Similarly, an earlier study suggested that elevated expression of histamine receptor H1 in HCC cells increased HCC cell growth and metastasis, whereas inactivation of histamine receptor H1 by terfenadine inhibited HCC tumor growth and metastasis.^25^ Interestingly, our data further showed that clemastine induced *CAT*, *SOD1*, and *TNFa* expression at 37°C. However, hyperthermia and clemastine exposure suppressed inflammatory signaling in OS cells. Clemastine induced expression of *IRE-1a* gene expression in U-2 OS and *ATF4* gene expression in Saos-2 at 37°C. No major changes in the expression of UPR biomarkers were observed in U-2 OS cells when cells were exposed to hyperthermia and clemastine. However, in Saos-2 cells, the expression of BIP and ATF6 was upregulated, suggesting that under hyperthermic conditions, clemastine modulates ER stress-related UPR signaling in OS cells. To further investigate the molecular mechanism of how clemastine and hyperthermic conditions affect cell proliferation and survival, we examined the expression of AKT/mTOR status in OS cells. Activation of the PI3K-AKT-mTOR pathway is known to enhance cell survival in various cancers including prostate cancer,^35^ liver cancer^36^ breast cancer,^37^ and OS.^38^ Interestingly, our data showed that exposure to clemastine alone did not suppress AKT/mTOR signaling. However, under hyperthermic conditions, clemastine reduced phosphorylation of S2448-mTOR and phosphorylation of S473-AKT suggesting that hyperthermic conditions and clemastine inactivate AKT/mTOR signaling in OS cells.

Inhibition of the PI3K/AKT/TOR pathway is known to induce autophagy in cancer cells.^39–41^ Autophagy is a cellular catabolic process involved in the elimination of unfolded proteins, and damaged cell organelles, through lysosomal degradation.^42^ Autophagy also regulates cell proliferation, differentiation, survival, and apoptosis, to maintain cellular homeostasis.^42^ Cellular autophagy is induced during nutrient depletion and participates in several cellular and developmental processes, including cell growth as well as programmed cell death/apoptosis.^43,44^ Several PI3K inhibitors, including LY294002, wortmannin, and 3-Methyladenine (an inhibitor of class III phosphoinositide 3-kinase) induce autophagy by inhibiting the PI3K/AKT/TOR pathway.^45–47^ Our data suggest that hyperthermic conditions inactivate AKT/mTOR and induce cellular autophagy in OS cells by inhibiting cellular ubiquitination since hyperthermic conditions inactivate ubiquitination.^48^ Hyperthermic conditions not only induced autophagy in OS cells but also triggered cell apoptosis as shown by increased expression of cleaved Caspase 3 and BAD expression. We further provided evidence that the inactivation of autophagy by 3-methyladenine inhibits hyperthermia and clemastine-mediated induction of LC3B II, LC3-positive puncta formation, and OS cell apoptosis. Inactivation of autophagy by 3-methyladenine did not affect OS cell survival when cells were exposed to clemastine and hyperthermia suggesting that clemastine and hyperthermia sensitized OS cell lines through autophagic cell death (Figure 6e).

In conclusion, we demonstrated that clemastine, an antagonist of histamine H1 receptor, under hyperthermia (42°C) conditions triggers cell death more efficiently than at 37°C. Hyperthermia-induced autophagy, and the combination of clemastine plus hyperthermia modulated cellular autophagic cell apoptosis. Therefore, our data suggest that hyperthermia along with chemotherapy may represent an improved approach to the treatment of OS.

## Materials and methods

### Cell culture and reagents preparation

The OS cell lines U-2 OS cells (Cat #HTB-96) and Saos-2 cells (Cat #HTB-85) were purchased from the American Type Culture Collection (ATCC Manassas, Virginia, USA). According to the recommendation of the supplier, the OS cells U-2 OS and Saos-2 were cultured in McCoy 5A medium (Fisher Scientific Cat #16600108) modified with L-glutamine and supplemented with 10% fetal bovine serum and 15% fetal bovine serum (FBS, Access Biologicals, Vista, CA), respectively. Cells were cultured in a 100 mm cell culture dish in a humidified incubator at 37°C supplied with 5% CO_2_. When cells reached 70–80% confluence they were used for experiments. Clemastine was purchased from Tocris Biosciences (Cat #1453100) and dissolved in DMSO. Autophagy inhibitor, 3-methyladenine (3-MA), was purchased from Thermo Scientific chemicals (Cat #AC379795000) and dissolved in DMSO.

### MTT assay

U-2 OS and Saos-2 cells (3 × 10^3^ cells/well) were seeded into 96 well plates and treated with vehicle or increasing concentrations (0.5 µM to 40 µM) of clemastine and/or 3-MA (2 mM) in a humidified incubator at 37°C for 72 h. Both cell lines containing various drug concentrations were then exposed to hyperthermic treatment by culturing in a 42°C incubator for 2 h and transferred back to a 37°C incubator to undergo the recovery phase for 6 h. After exposure, cells were then incubated with 5 µl/well of MTT (3-(4,5-dimethylthiazol-2-yl)-2,5-diphenyltetrazolium bromide) reagent (stock 5 mg/ml in PBS) for 30 min at 37°C in a cell culture incubator. Cells were then carefully washed with PBS, and formazan crystals were dissolved in 100 µl DMSO. Measurement of cell survival was carried out by reading the plates at 570 nm using a Fluostar Omega plate reader (BMG Lab tech, Cary, NC). The experiments were performed in triplicates.

### Cell colony formation assay

U-2 OS and Saos-2 cells (10,000/well) were seeded into 6-well plates in triplicates for 24 h then exposed with vehicles or clemastine (10 µM) in a humidified incubator at 37°C for 72 h. Both cell lines were then exposed to hyperthermic treatment by exposing the OS cells to a 42°C incubator for 2 h and transferred back to a 37°C incubator to undergo the recovery phase for 72 h. Cells were washed with PBS, fixed with cold methanol, and stained with 0.5% crystal violet for 30 min. Cells were washed with distilled water and allowed to dry. Blue colonies were photographed, counted manually, and plotted.

### ATP assay

Cell viability was measured by ATP assay using the Cell-Titer Glo assay kit (Promega Corporation, USA). U-2 OS cells were seeded on 96-well plates and then treated with clemastine and cultured in a humidified incubator at 37°C for 72 h. Then, the cells (aside from a control set) were exposed to hyperthermic treatment by incubating in a 42°C incubator for 2 h and transferred back to the 37°C incubator to recover overnight. The Cell-Titer Glo buffer and Cell-Titer Glo substrate were thawed in a 37°C water bath, and 100 μl was added to each well in the 96-well plates. Plates were wrapped in foil to avoid light penetration and then kept on a shaker for 20 min to stabilize luminescence signals. The luminescence was then recorded, and the results were analyzed.

### Seahorse bioanalyzer

The Cell Mito-Stress Assay was used to characterize the effect of clemastine + hyperthermia on mitochondrial oxygen consumption rate (OCR). OS cells were exposed to clemastine + hyperthermia as indicated, and cells (2 × 10^4^ cells/well) were seeded in XFp plates with XF media and supplements as described in the manufacturer’s instructions for the XF Cell Mito-Stress assay. Oxygen consumption rate (OCR) was measured using a Seahorse XFp analyzer. For OS cells, 1 μM Oligomycin, 0.5 μM FCCP, and 0.5 μM Rotenone/antimycin A were simultaneously applied to measure OCR in the cells. The results were analyzed using Wave software (Seahorse/Agilent).

### Spheroid formation assay

U-2 OS and Saos-2 cells (10,000/well) were seeded into 24 well ultra-low attachment surface plates for 24 h and exposed to vehicles or clemastine (10 µM) for 72 h. Cells were then exposed to hyperthermic treatment by incubating the cells at 42°C for 2 h and transferred back to a 37°C incubator to undergo the recovery phase for 48 h. U-2 OS and Saos-2 cell spheroid formation were monitored, and images were captured under a light microscope using a 4X objective. The spheroid area (*n* = 3) was calculated using ImageJ software (https://imagej.nih.gov/ij/) and plotted.

### Western blotting

U-2 OS and Saos-2 cells were cultured on a 100 mm dish and treated with vehicle and/or clemastine for 72 h then exposed to hyperthermic treatment by culturing in a 42°C incubator for 2 h and returned to a 37°C incubator for 6 h recovery phase. The OS cells were washed with cold PBS and lysed in cell lysis buffer (Cell Signaling Technology, Danvers, MA) containing a protease inhibitor cocktail (Roche, Indianapolis, IN). The cell lysates were prepared by centrifugation at 8000 rpm for 15 min, and the supernatants were collected and used for protein quantification. To determine the protein concentrations, a Bio-Rad protein assay reagent (Bio-Rad, Hercules, CA) was used. For immunoblotting, 60 µg of protein lysates were separated by using NuPAGE 4–12% Bis-Tris-SDS gels (Invitrogen). The proteins were transferred to polyvinylidene difluoride (PVDF) membranes (Millipore, Billerica, MA). After transferring for 1½ h, the membranes were washed with 1X Tris-buffered saline with 0.1% Tween 20 (TBS-T) and blocked in 1X blocking buffer (Sigma-Aldrich, St. Louis, MO) for 15 min before incubating them with primary antibodies (1:1000 dilution) as instructed in manufacturer’s protocol on a shaker overnight at −4°C. The following antibodies were obtained from Cell Signaling Technology (Danvers, MA): anti-cleaved caspase 3 (Cat #9664S), anti-caspase 3 (Cat #9662S), anti-parp (Cat #9542P) anti-cleaved parp (Cat # 9541S), anti-β-tubulin (Cat #2128S), anti-SOD1 (Cat #2770S), anti-CAT (#12980T), anti-BIP (Cat #3177P), anti-pERK (Cat #5683P), anti-p-elf2α (Cat #3398P), anti-Ire1α (Cat #3294S), anti-GAPDH (Cat #2118S), anti-pS473-AKT (Cat #3787S), anti-AKT (Cat # 9272S), anti-pS2448-mTOR (Cat #2971S), anti-mTOR (Cat # 2983S), anti-SIRT1 (Cat #2028S), anti-Beclin1 (Cat #3738S), anti-RAB7 (9367S), anti-LAMP1 (Cat #9091S), anti-p62 (Cat #5114S), anti-LC3B I/II (Cat #43566S), anti-BAD (Cat #9292S), anti-BCL-xL (Cat #2764S), anti-Survivin (Cat #2808S), and anti-β-actin (Cat #4970S). We also purchased anti-ATF6 (SC-22799) and anti-ubiquitin (Cat #sc -53,309), from Santa Cruz Biotechnology (Dallas, TX). After incubating overnight, the western blots were washed for 5 min three times with TBS-T and incubated for 1½ h with the appropriate anti-rabbit or anti-mouse secondary antibody (dilution ratio of 1:10000). This was performed at room temperature, and the anti-mouse/anti-rabbit secondary antibody was purchased from Jackson ImmunoResearch, PA. ECL solution (ThermoScientific, Cat #34580) was used to develop the immunoblots which were then visualized using the Azure instrument (C-500 Bio-system). Immunoblots were repeated three times, and one set of data was presented. Band intensities were quantified by Image J (https://imagej.nih.gov/ij/) and plotted.

### RT/qPCR

U-2 OS and Saos-2 cells were cultured in six-well plates and treated with vehicle and/or clemastine for 72 h then exposed to hyperthermic treatment by culturing in a 42°C incubator for 2 h before being returned to a 37°C incubator for 6 h recovery phase. The OS cells were washed with cold PBS, and total RNA was isolated using TRIZOL reagent (Invitrogen, Carlsbad, CA). RNA (1 µg) was reverse transcribed using a High Capacity cDNA Reverse Transcription kit (Applied Biosystems, Carlsbad, CA), and cDNA was mixed with Power SYBR Green PCR master mix (Applied Biosystems) with forward and reverse specific primers as indicated (Supplementary Table S1). GAPDH forward and reverse primers were used as an internal control. The PCR mixtures were run on a QuantStudio-3 PCR System (Applied Biosystems) using relative quantitation according to the manufacturer’s protocols.

### Transient transfection and LC3 puncta assessment

U-2 OS and Saos-2 (1 × 10^5^ cells) were grown on coverslips in 6-well plates for 24 h and transfected with 1 μg of EGFP-LC3B plasmid DNA and cells were treated with vehicle and/or clemastine for 60 h then exposed to hyperthermic treatment by culturing in 42°C incubator for 2 h before being returned to 37°C incubator for 6 h recovery phase. In another experiment, U-2 OS and Saos-2 (1 × 10^5^ cells) were grown on coverslips in 6-well plates for 24 h and co-transfected with 1 μg of EGFP-LC3B plasmid DNA and cells were treated with vehicle or clemastine for 1 h then treated with 2 mM 3-MA for 60 h and exposed to hyperthermic treatment by culturing in 42°C incubator for 2 h before being returned to 37°C incubator for 6 h recovery phase. Cells were washed with PBS and fixed with 4% paraformaldehyde for 15 min. Cells were washed twice with PBS and mounted with Vectashield mounting medium (Vector Lab.) containing nuclear DAPI stain. Cells were imaged using a ZEISS LSM 800 Confocal microscope fluorescent microscope. The number of GFP-LC3B-related puncta formed in the cells (*n* = 4) was counted.

### Statistical analysis

Results from independent triplicate experiments are presented as mean ± SEM. Differences between groups were analyzed using a two-tailed Student’s *t*-test. Statistical significance between means was determined by Graph Pad Prism 9 software (GraphPad Software Inc., La Jolla, CA). A *p*-value of <0.05 was considered statistically significant.

## Highlights

1. Hyperthermia sensitizes osteosarcoma cells to clemastine-mediated cell apoptosis.
2. Clemastine and hyperthermia modulate inflammatory and unfolded protein response (UPR) signaling in osteosarcoma cells.
3. Exposure to clemastine and hyperthermia inhibits AKT/mTOR signaling and induces autophagy biomarkers.
4. Clemastine and hyperthermia exposure sensitizes osteosarcoma cells for autophagic cell death.

## Supplementary Material

Supplemental Material

## Data Availability

The published article includes all data sets generated/analyzed for this study. Data will be made available on request.

## Abbrevations

OS: Osteosarcoma
U-2 OS: Human Osteosarcoma cell line
Saos-2: Human Osteosarcoma cell line
MG-63: Human Osteosarcoma cell line
miRNAs: Micro- Ribonucleic acid
miR-21-5p: a type of micro RNA
CT-Scan: Computed tomography scan
MSKCC: Memorial Sloan-Kettering Cancer Center
MAP: Mitogen-activated Protein Kinase
p53: Tumor suppression gene
C-MYC: Cellular Myelocytomatosis
MCF-7: Michigan Cancer Foundation-7 breast cancer cells
ER: Endoplasmic Reticulum
ATP: Adenosine Triphosphate
HIF-1α: Hypoxia-Inducible Factor 1 alpha
VEGF: Vascular Endothelial Growth Factor
GEM: Gemcitabine
PANC-1 & ASPC-1: Pancreatic cancer cells
STS: Soft-tissue Sarcoma
AMF: Autocrine Motility Factor
H1R: Histamine 1 Receptor
H2R: Histamine 2 Receptor
H3R: Histamine 3 receptor
H4R: Histamine 4 receptor
HCC: Hepatocellular Carcinoma cells
BAD: Proapoptotic BH3-only protein
TNFAIP8: Tumor Necrosis Factor alpha-induced Protein 8
FBS: Fetal Bovine Serum
DMSO: Dimethyl sulfoxide
3-MA: 3-methyladenine
PBS: Phosphate Buffer saline
PVDF: Polyvinylidene Difluoride
SOD1: Superoxide Dismutase 1
BIP: Binding Immunoglobin protein
PERK: RNA-like Endoplasmic reticulum Kinase
P-elf2 α: Phosphorylated Eukaryotic Initiation factor 2 alpha
SIRT1: Sirtuin 1
RAB 7: Ras-related protein
P-mTOR: Phosphorylated mammalian target of rapamycin
LAMP1: Lysosomal associated membrane protein1
P62: Ubiquitin binding protein 62
LC3B: Microtubule-associated protein 1- Light Chain 3 Beta
ATF6: Activating Transcription Factor 6
CSC: Cancer Stem cells

## References

[1] Mirabello L, Troisi RJ, Savage SA. International osteosarcoma incidence patterns in children and adolescents, middle ages and elderly persons. Int J Cancer. 2009;125(1):229–13. doi:10.1002/ijc.24320. 19330840 PMC3048853

[2] Simon MA, Aschliman MA, Thomas N, Mankin HJ. Limb-salvage treatment versus amputation for osteosarcoma of the distal end of the femur. J Bone Joint Surg Am. 1986;68(9):1331–1337. doi:10.2106/00004623-198668090-00005. 3465732

[3] Misaghi A, Goldin A, Awad M, Kulidjian AA. Osteosarcoma: a comprehensive review. Sicot J. 2018;4:12. doi:10.1051/sicotj/2017028. 29629690 PMC5890448

[4] Bicher HI, Hetzel FW, Sandhu TS, Frinak S, Vaupel P, O’Hara MD, O’Brien T. Effects of hyperthermia on normal and tumor microenvironment. Radiol. 1980;137(2):523–530. doi:10.1148/radiology.137.2.7433686. 7433686

[5] Cheng Y, Weng S, Yu L, Zhu N, Yang M, Yuan Y. The role of hyperthermia in the multidisciplinary treatment of malignant tumors. Integr Cancer Ther. 2019;18:1534735419876345. doi:10.1177/1534735419876345. 31522574 PMC7242805

[6] Fajardo LF, Schreiber AB, Kelly NI, Hahn GM. Thermal sensitivity of endothelial cells. Radiat Res. 1985;103(2):276–285. doi:10.2307/3576582. 4023180

[7] Hurwitz M, Stauffer P. Hyperthermia, radiation and chemotherapy: the role of heat in multidisciplinary cancer care. Semin Oncol. 2014;41(6):714–729. doi:10.1053/j.seminoncol.2014.09.014. 25499632

[8] Horseman M, Panahi L, Udeani G, Tenpas AS, Verduzco R Jr., Patel PH, Bazan DZ, Mora A, Samuel N, Mingle AC. et al. Drug-induced hyperthermia review. Cureus. 2022;14:e27278. doi:10.7759/cureus.27278. 36039261 PMC9403255

[9] Imashiro C, Jin Y, Hayama M, Yamada TG, Funahashi A, Sakaguchi K, Umezu S, Komotori J. Titanium culture vessel presenting temperature gradation for the thermotolerance estimation of cells. Cyborg Bionic Syst. 2023;4:0049. doi:10.34133/cbsystems.0049. 37554432 PMC10405790

[10] Maduabuchi WO, Tansi FL, Heller R, Hilger I. Hyperthermia influences the secretion signature of tumor cells and affects endothelial cell sprouting. Biomed. 2023;11(8):2256. doi:10.3390/biomedicines11082256. PMC10452125 37626752

[11] Xi P, Ma X, Hu F, Li L, Liu H, Zhou J, Wu W. ROS-Sp1 axis is involved in thermochemotherapy-enhanced sensitivity of pancreatic cancer cells to gemcitabine. Cell Biol Int. 2023;47(11):1825–1834. doi:10.1002/cbin.12073. 37545170

[12] Hiramoto RN, Ghanta VK, Lilly MB. Reduction of tumor burden in a murine osteosarcoma following hyperthermia combined with cyclophosphamide. Cancer Res. 1984;44:1405–1408. 6584205

[13] Tancredi A, Ciuffreda L, Cuttitta A, Scaramuzzi R, Sabatino R, Scaramuzzi G. Hyperthermia in the treatment of post-actinic osteosarcomas: our anecdotal experience. Eurasian J Med. 2011;43(2):115–118. doi:10.5152/eajm.2011.25. 25610175 PMC4261353

[14] Reinhold HS, Endrich B. Tumour microcirculation as a target for hyperthermia. Int J Hyperthermia. 1986;2(2):111–137. doi:10.3109/02656738609012389. 3540146

[15] Issels RD, Lindner LH, Verweij J, Wust P, Reichardt P, Schem BC, Abdel-Rahman S, Daugaard S, Salat C, Wendtner CM. et al. Neo-adjuvant chemotherapy alone or with regional hyperthermia for localised high-risk soft-tissue sarcoma: a randomised phase 3 multicentre study. Lancet Oncol. 2010;11(6):561–570. doi:10.1016/S1470-2045(10)70071-1. 20434400 PMC3517819

[16] Alcaide M, Ramirez-Santillan C, Feito MJ, de la Concepción Matesanz M, Ruiz-Hernandez E, Arcos D, Vallet-Regi M, Portoles MT. In vitro evaluation of glass–glass ceramic thermoseed-induced hyperthermia on human osteosarcoma cell line. J Biomed Mater Res A. 2012;100A(1):64–71. doi:10.1002/jbm.a.33229. 21972012

[17] Trieb K, Blahovec H, Kubista B. Effects of hyperthermia on heat shock protein expression, alkaline phosphatase activity and proliferation in human osteosarcoma cells. Cell Biochem Funct. 2007;25(6):669–672. doi:10.1002/cbf.1371. 16933368

[18] Shui C, Scutt A. Mild heat shock induces proliferation, alkaline phosphatase activity, and mineralization in human bone marrow stromal cells and Mg-63 cells in vitro. J Bone Miner Res. 2001;16(4):731–741. doi:10.1359/jbmr.2001.16.4.731. 11316001

[19] Hou CH, Lin FL, Hou SM, Liu JF. Hyperthermia induces apoptosis through endoplasmic reticulum and reactive oxygen species in human osteosarcoma cells. Int J Mol Sci. 2014;15(10):17380–17395. doi:10.3390/ijms151017380. 25268613 PMC4227168

[20] Nakajima K, Yanagawa T, Watanabe H, Takagishi K. Hyperthermia reduces migration of osteosarcoma by suppression of autocrine motility factor. Oncol Rep. 2012;28(6):1953–1958. doi:10.3892/or.2012.2066. 23027359 PMC3583516

[21] Shido Y, Nishida Y, Suzuki Y, Kobayashi T, Ishiguro N. Targeted hyperthermia using magnetite cationic liposomes and an alternating magnetic field in a mouse osteosarcoma model. J Bone Joint Surg Br. 2010;92-B(4):580–585. doi:10.1302/0301-620X.92B4.22814. 20357339

[22] Seynhaeve ALB, Amin M, Haemmerich D, van Rhoon GC, Ten Hagen TLM. Hyperthermia and smart drug delivery systems for solid tumor therapy. Adv Drug Deliv Rev. 2020;163-164:125–144. doi:10.1016/j.addr.2020.02.004. 32092379

[23] Nair MP, Schwartz SA. Effect of histamine and histamine antagonists on natural and antibody-dependent cellular cytotoxicity of human lymphocytes in vitro. Cell Immunol. 1983;81(1):45–60. doi:10.1016/0008-8749(83)90210-1. 6225527

[24] Nguyen PL, Cho J. Pathophysiological roles of histamine receptors in cancer progression: implications and perspectives as potential molecular targets. Biomolecul. 2021;11(8):1232. doi:10.3390/biom11081232. PMC8392479 34439898

[25] Zhao J, Hou Y, Yin C, Hu J, Gao T, Huang X, Zhang X, Xing J, An J, Wan S. et al. Upregulation of histamine receptor H1 promotes tumor progression and contributes to poor prognosis in hepatocellular carcinoma. Oncogene. 2020;39(8):1724–1738. doi:10.1038/s41388-019-1093-y. 31740780 PMC7033043

[26] Nakano K. Challenges of systemic therapy investigations for bone sarcomas. Int J Mol Sci. 2022;23(7):3540. doi:10.3390/ijms23073540. 35408900 PMC8998654

[27] Peng Y, Wang Y, Zhou C, Mei W, Zeng C. PI3K/Akt/mTOR pathway and its role in cancer therapeutics: are we making headway? Front Oncol. 2022;12:819128. doi:10.3389/fonc.2022.819128. 35402264 PMC8987494

[28] Varshney P, Saini N. PI3K/AKT/mTOR activation and autophagy inhibition plays a key role in increased cholesterol during IL-17A mediated inflammatory response in psoriasis. Biochim Biophys Acta Mol Basis Dis. 2018;1864(5):1795–1803. doi:10.1016/j.bbadis.2018.02.003. 29432814

[29] Heckmann BL, Yang X, Zhang X, Liu J. The autophagic inhibitor 3-methyladenine potently stimulates PKA-dependent lipolysis in adipocytes. Br J Pharmacol. 2013;168:163–171. doi:10.1111/j.1476-5381.2012.02110.x. 22817685 PMC3570012

[30] Biermann JS, Adkins DR, Agulnik M, Benjamin RS, Brigman B, Butrynski JE, Cheong D, Chow W, Curry WT, Frassica DA. et al. Bone cancer. J Natl Compr Canc Netw. 2013;11(6):688–723. doi:10.6004/jnccn.2013.0088. 23744868

[31] Lindsey BA, Markel JE, Kleinerman ES. Osteosarcoma overview. Rheumatol Ther. 2017;4(1):25–43. doi:10.1007/s40744-016-0050-2. 27933467 PMC5443719

[32] Hughes DP. Strategies for the targeted delivery of therapeutics for osteosarcoma. Expert Opin Drug Deliv. 2009;6(12):1311–1321. doi:10.1517/17425240903280422. 19761419 PMC4163784

[33] Bacci G, Longhi A, Bertoni F, Briccoli A, Versari M, Pignotti E, Picci P. Bone metastases in osteosarcoma patients treated with neoadjuvant or adjuvant chemotherapy the Rizzoli experience in 52 patients. Acta Orthop. 2006;77(6):938–943. doi:10.1080/17453670610013268. 17260205

[34] PosthumaDeboer J, Witlox MA, Kaspers GJ, van Royen BJ. Molecular alterations as target for therapy in metastatic osteosarcoma: a review of literature. Clin Exp Metastasis. 2011;28(5):493–503. doi:10.1007/s10585-011-9384-x. 21461590 PMC3081058

[35] Pungsrinont T, Kallenbach J, Baniahmad A. Role of PI3K-AKT-mTOR pathway as a pro-survival signaling and resistance-mediating mechanism to therapy of prostate cancer. Int J Mol Sci. 2021;22(20):11088. doi:10.3390/ijms222011088. 34681745 PMC8538152

[36] Tian LY, Smit DJ, Jucker M. The role of PI3K/AKT/mTOR signaling in hepatocellular carcinoma metabolism. Int J Mol Sci. 2023;24(3):2652. doi:10.3390/ijms24032652. 36768977 PMC9916527

[37] Paplomata E, O’Regan R. The PI3K/AKT/mTOR pathway in breast cancer: targets, trials and biomarkers. Ther Adv Med Oncol. 2014;6(4):154–166. doi:10.1177/1758834014530023. 25057302 PMC4107712

[38] Ding L, Congwei L, Bei Q, Tao Y, Ruiguo W, Heze Y, Bo D, Zhihong L. mTOR: an attractive therapeutic target for osteosarcoma? Oncotarget. 2016;7(31):50805–50813. doi:10.18632/oncotarget.9305. 27177330 PMC5226621

[39] He C, Klionsky DJ. Regulation mechanisms and signaling pathways of autophagy. Annu Rev Genet. 2009;43(1):67–93. doi:10.1146/annurev-genet-102808-114910. 19653858 PMC2831538

[40] Kim KW, Mutter RW, Cao C, Albert JM, Freeman M, Hallahan DE, Lu B. Autophagy for cancer therapy through inhibition of pro-apoptotic proteins and mammalian target of rapamycin signaling. J Biol Chem. 2006;281:36883–36890. doi:10.1074/jbc.M607094200. 17005556

[41] Verschooten L, Barrette K, Van Kelst S, Rubio Romero N, Proby C, De Vos R, Agostinis P, Garmyn M, Koul H. Autophagy inhibitor chloroquine enhanced the cell death inducing effect of the flavonoid luteolin in metastatic squamous cell carcinoma cells. PLOS ONE. 2012;7(10):e48264. doi:10.1371/journal.pone.0048264. 23110223 PMC3482182

[42] Yorimitsu T, Klionsky DJ. Autophagy: molecular machinery for self-eating. Cell Death Differ. 2005;12(Suppl S2):1542–1552. doi:10.1038/sj.cdd.4401765. 16247502 PMC1828868

[43] O’Farrell F, Wang S, Katheder N, Rusten TE, Samakovlis C, Nusse R. Two-tiered control of epithelial growth and autophagy by the insulin receptor and the ret-like receptor, stitcher. PLOS Biol. 2013;11(7):e1001612. doi:10.1371/journal.pbio.1001612. 23935447 PMC3720245

[44] Scott RC, Juhasz G, Neufeld TP. Direct induction of autophagy by Atg1 inhibits cell growth and induces apoptotic cell death. Curr Biol. 2007;17(1):1–11. doi:10.1016/j.cub.2006.10.053. 17208179 PMC1865528

[45] Rodon J, Dienstmann R, Serra V, Tabernero J. Development of PI3K inhibitors: lessons learned from early clinical trials. Nat Rev Clin Oncol. 2013;10(3):143–153. doi:10.1038/nrclinonc.2013.10. 23400000

[46] Arcaro A, Wymann MP. Wortmannin is a potent phosphatidylinositol 3-kinase inhibitor: the role of phosphatidylinositol 3,4,5-trisphosphate in neutrophil responses. Biochem J. 1993;296(2):297–301. doi:10.1042/bj2960297. 8257416 PMC1137693

[47] Wu YT, Tan HL, Shui G, Bauvy C, Huang Q, Wenk MR, Ong CN, Codogno P, Shen HM. Dual role of 3-methyladenine in modulation of autophagy via different temporal patterns of inhibition on class I and III phosphoinositide 3-kinase. J Biol Chem. 2010;285:10850–10861. doi:10.1074/jbc.M109.080796. 20123989 PMC2856291

[48] Aliabadi F, Sohrabi B, Mostafavi E, Pazoki-Toroudi H, Webster TJ. Ubiquitin–proteasome system and the role of its inhibitors in cancer therapy. Open Biol. 2021;11(4):200390. doi:10.1098/rsob.200390. 33906413 PMC8080017

