# Supplementary material for: Clemastine and hyperthermia enhance sensitization of osteosarcoma cells for apoptosis
Source: Mol Cell Oncol. 2024 May 14;11(1):2351622. doi: 10.1080/23723556.2024.2351622 (PMC11110698; doi:10.1080/23723556.2024.2351622)
Supplement: Supplemental Material [file KMCO_A_2351622_SM7068.zip › Supp_f___t-NEW/Supplementary_figure_legends.docx]

**Supplementary Figure legends**

**Fig. S1:** **Cell viability assay.** U-2 OS and Saos-2 cells (1x10^5^) were plated in 6 well plates for 24h and treated with 6 µM clemastine for 72h. The hyperthermic treatment was performed in a 42°C incubator for 2h and returned to a 37°C incubator for 6hrs recovery phase. Cells were stained with trypan blue and viable cell numbers were quantified by TC-20 automated cell counter (Bio-Rad) **P*<0.05, ***P*<0.01, ****P*<0.001 compared to the vehicle untreated cells.

**Fig. S2: Mitochondrial oxygen consumption rate (OCR) analysis.** U-2 OS and Saos-2 cells (1x10^5^) were plated in 6 well plates for 24h and treated with 6 µM clemastine for 72h. The hyperthermic treatment was performed in a 42°C incubator for 2h and returned to a 37°C incubator for 6 h recovery phase as indicated. Cells (2 x 10^4^ cells/well) were subjected to Seahorse Bio-analyzer, and relative OCR was analyzed as described in the materials and methods section. O- Oligomycin. F- FCCP. R/A- Rotenone and Antimycin. ns- not significant.

**Fig. S3: Cleaved caspase 3 and cleaved PARP quantification.** Immunoblot band intensities were quantified by Image J (<https://imagej.nih.gov/ij/>) and plotted. **P*<0.05 compared to untreated cells and exposed at 37°C.

**Fig. S4: Effect of 3-MA on mitochondrial OCR.** U-2 OS and SAOS-2 cells were treated 3-MA or 3-MA+clemastine (6µM) for 72h. The hyperthermic treatment was performed in a 42°C incubator for 2h and then returned to 37°C for a 6h recovery phase. Cells (2 x 10^4^ cells/well) were subjected to Seahorse Bio-analyzer, and relative OCR was analyzed as described in the materials and methods section. O- Oligomycin. F- FCCP. R/A- Rotenone and Antimycin. ns- not significant.
