# Supplementary material for: Clemastine and hyperthermia enhance sensitization of osteosarcoma cells for apoptosis
Source: Mol Cell Oncol. 2024 May 14;11(1):2351622. doi: 10.1080/23723556.2024.2351622 (PMC11110698; doi:10.1080/23723556.2024.2351622)
Supplement: Supplemental Material [file KMCO_A_2351622_SM7068.zip › Supp_f___t-NEW/Table S1.docx]

**Supplementary Table 1**

**Set of primers used in the current study.**

| Primer sequence |
| --- |
| GAPDH FP: 5’-CCACCCAGAAGACTGTGGAT -3’  GAPDH RP: 5’-GTTGAAGTCAGAGGAGACCACC-3’ |
| IRE1a FP: 5’-GTGGCCTTCATCATCACCTATC-3’  IRE1a RP: 5’-CCTCATCTCCATCGTCTTGTTC-3’ |
| eIF2a FP: 5’-CCTTCTGCTGCCTCTAAGATAAA-3’  eIF2a RP: 5’-GGGAGCTGAGTGCTACAATAAA-3’ |
| ATF4 FP: 5’-CCATGATCCCTCAGTGCATAA-3’  ATF4 RP: 5’-TGCGGACCTCTTCTATCAAATC-3’ |
| BIP FP: 5’-CCTTCGATGTGCCTCTTCTCAC-3’  BIP RP: 5’-GGACGGGCTTCATAGTA-3’ |
| ATF6 FP: 5’-GAACTTCGAGGATGGGTTCATAG-3’  ATF6 RP: 5’-GTGGTCTTGTTATGGGTGGTGGTAG-3’ |
| CAT FP: CCTCTCATCCCAGTTGGTAAAC  CAT RP: TGTTGAATCTCCGCACTTCTC |
| SOD1FP: GTGTGGCCGATGTGTCTATT  SOD1RP CTCAGACTACATCCAAGGGAATG |
| CXCL8(IL8) FP: AGACAGCAGAGCACACAAG  CXCL8(IL8) RP: GGGTGGAAAGGTTTGGAGTAT |
| IL6 FP: 5’-GTAGTGAGGAACAAGCCAGAG-3’  IL6 RP: 5’-GGACTGCAGGAACTCCTTAAA-3’ |
| TNFα FP: 5’- GAGCCAGCTCCCTCTATTTATG-3’  TNFα RP: 5’- AGGGCGATTACAGACACAAC-3’ |
| TNFAIP8 FP: 5’- CTTTGACCGGAATGTGTTATCCA-3’  TNFAIP8 FP: 5’ - CAAGGCAGCCAAAAATTCACAA-3’ |
| p62 FP: 5’-GGA ACA GAT GGA GTC GGA TAA C-3’  p62 RP: 5’-CTG GAA GAA GGC AGA GAA ACT-3’ |
| LC3b FP: 5’-CCT AAG AGA ACC ACA CCC AAA G-3’  LC3b RP: 5’-CAG AAG GGA GTG TGT CTG AAT G-3’ |
| BECLN FP: 5’-TCA GGA GGA AGC TCA GTA TCA-3’  BECLN RP: 5’-CCG TAA GGA ACA AGT CGG TAT C-3’ |
| ATG3 FP: 5’-CCG GTC CTC AAG GAA TCA AA-3’  ATG3 RP: 5’-CGC CAT CAC CAT CAT CTT CT-3’ |
| VPS34 FP: 5’-GAC GGA AGA TGG AGG CAA ATA-3’  VPS34 RP: 5’-CCA CAG GAA CTG ACT GGA TAA A-3’ |
