# Supplementary figures and images for: Clemastine and hyperthermia enhance sensitization of osteosarcoma cells for apoptosis
Source: Mol Cell Oncol. 2024 May 14;11(1):2351622. doi: 10.1080/23723556.2024.2351622 (PMC11110698; doi:10.1080/23723556.2024.2351622)

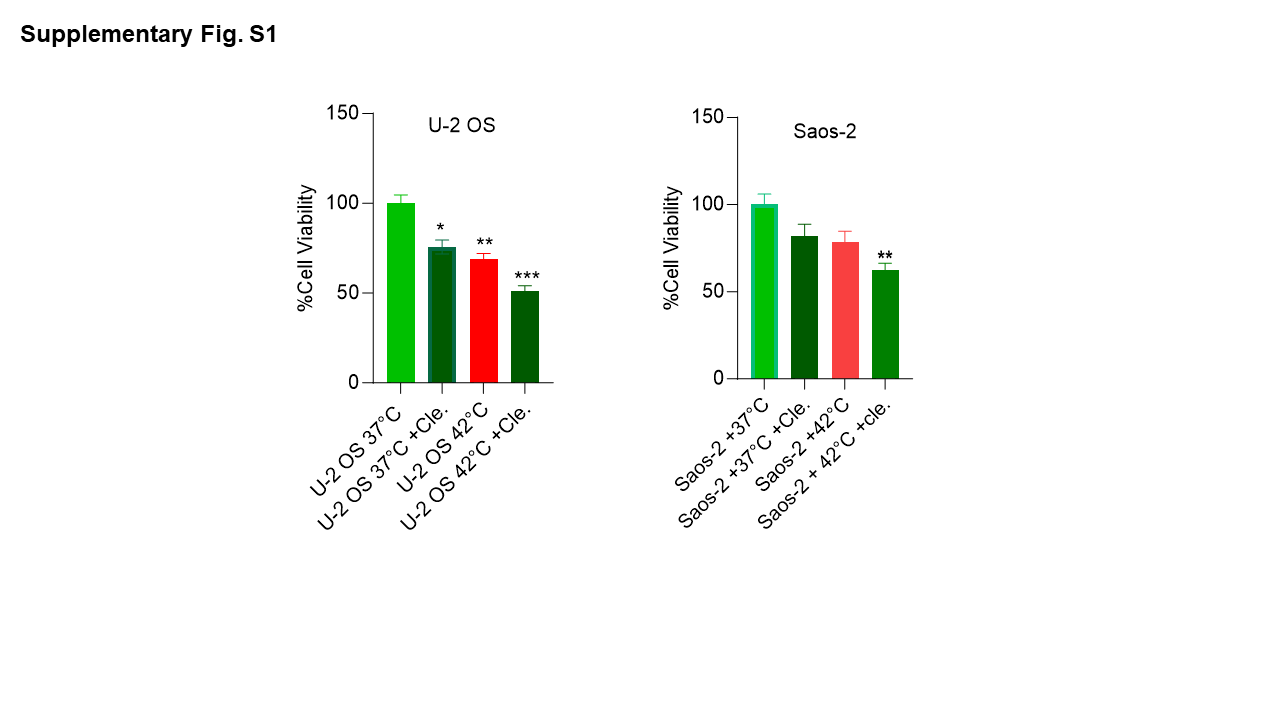

Supplement: Supplemental Material [file KMCO_A_2351622_SM7068.zip › Supp_f___t-NEW/FigS 1.tif]

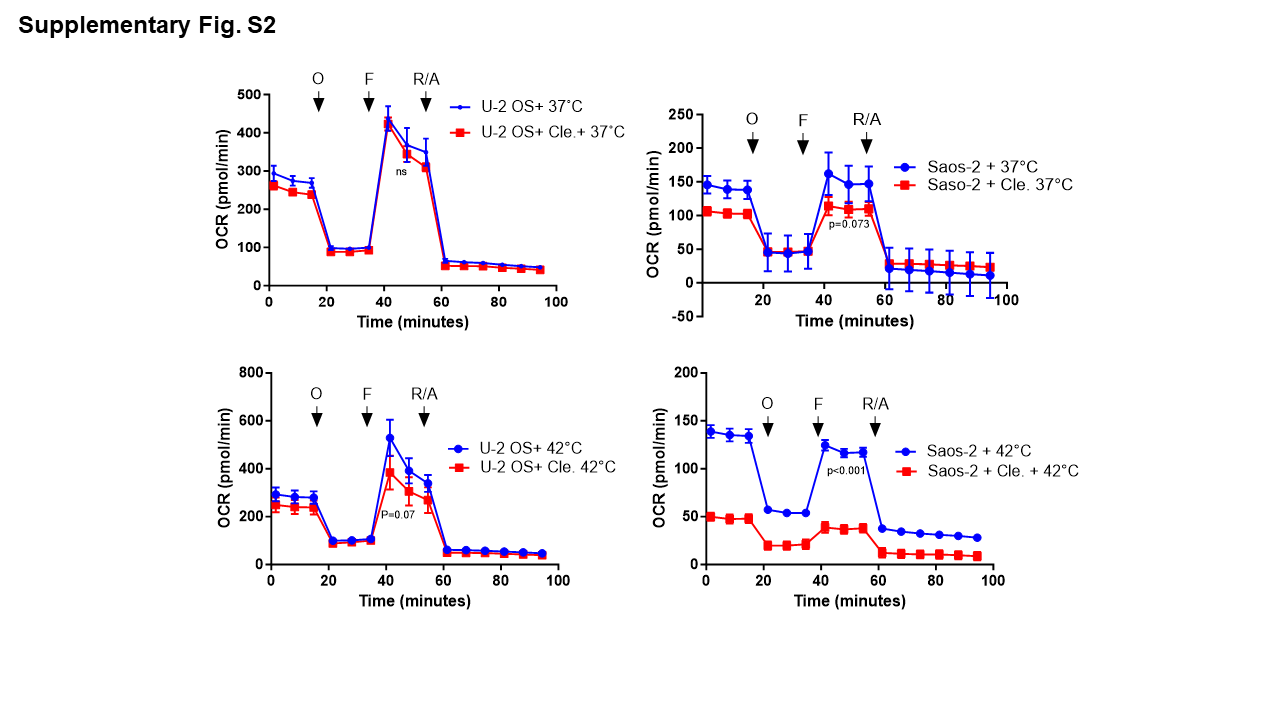

Supplement: Supplemental Material [file KMCO_A_2351622_SM7068.zip › Supp_f___t-NEW/FigS 2.tif]

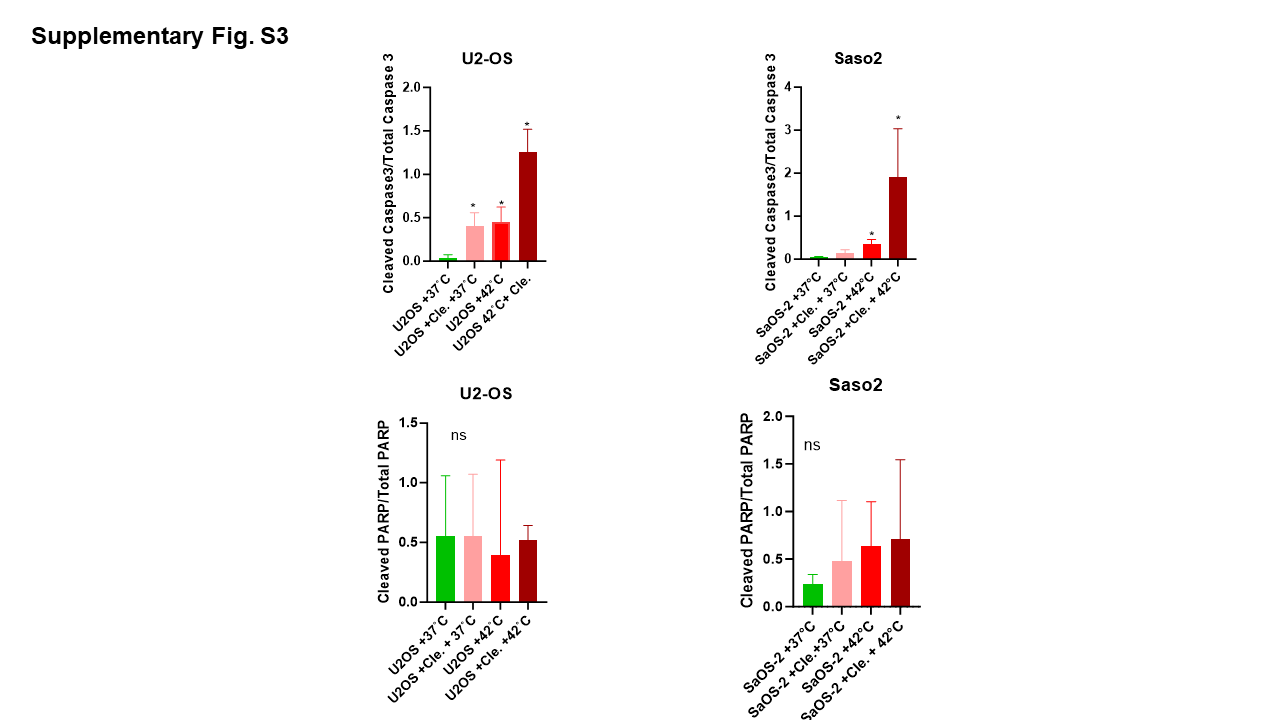

Supplement: Supplemental Material [file KMCO_A_2351622_SM7068.zip › Supp_f___t-NEW/FigS 3.tif]

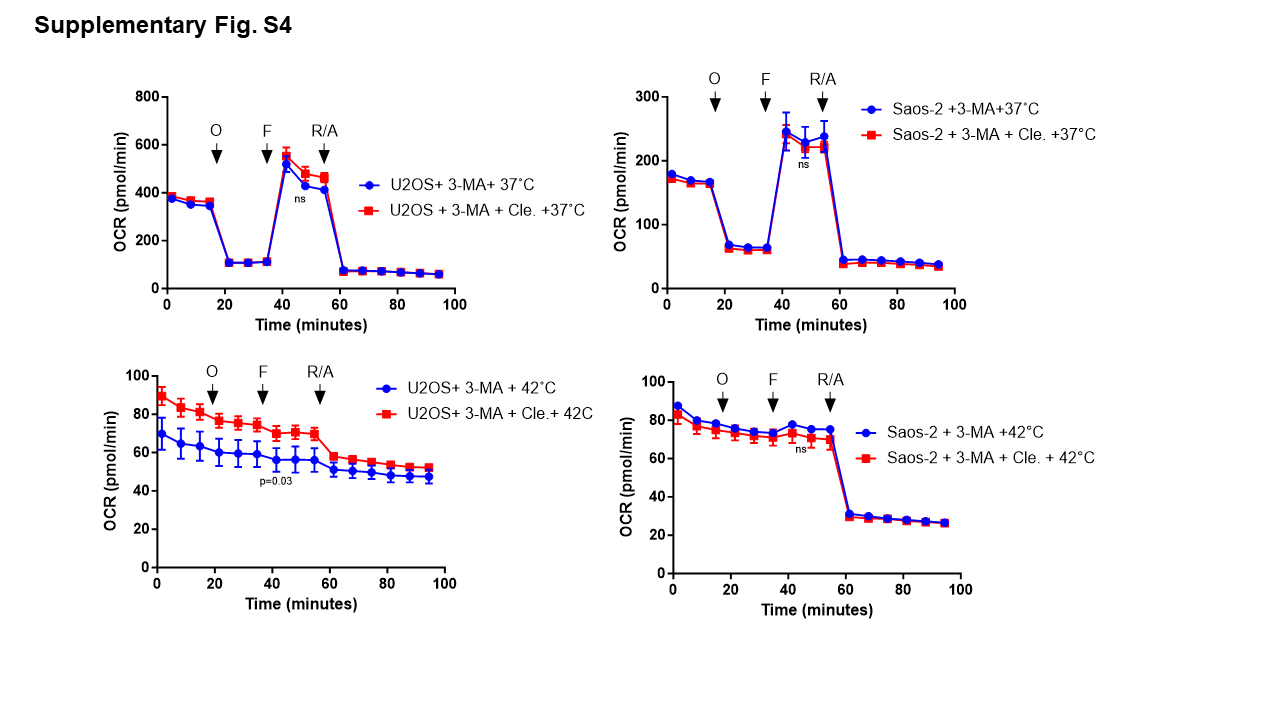

Supplement: Supplemental Material [file KMCO_A_2351622_SM7068.zip › Supp_f___t-NEW/FigS 4.tif]
